# Survival, growth, and biogenic amine production of *Enterococcus faecium* FC12 in response to extracts and essential oils of *Rubus fruticosus* and *Juniperus oxycedrus*

**DOI:** 10.3389/fnut.2022.1092172

**Published:** 2023-01-13

**Authors:** Chiara Montanari, Federica Barbieri, Silvia Lorenzini, Davide Gottardi, Vida Šimat, Fatih Özogul, Fausto Gardini, Giulia Tabanelli

**Affiliations:** ^1^Department of Agricultural and Food Sciences, University of Bologna, Cesena, Italy; ^2^Interdepartmental Center for Industrial Agri-food Research, University of Bologna, Cesena, Italy; ^3^University Department of Marine Studies, University of Split, Split, Croatia; ^4^Department of Seafood Processing Technology, Faculty of Fisheries, Cukurova University, Adana, Turkey; ^5^Department of Agricultural and Food Sciences, University of Bologna, Bologna, Italy

**Keywords:** *Enterococcus faecium*, *Juniperus oxycedrus*, *Rubus fruticosus*, antimicrobial activity, growth kinetics, flow cytometry, tyramine production

## Abstract

**Introduction:**

Enterococci are lactic acid bacteria (LAB) usually found as food contaminants in fermented products such as cheeses and fermented sausages. Due to their antibiotic resistance, the presence of virulence factors, and the ability to produce biogenic amines (BAs), the determination of these bacteria is crucial to assure food quality and safety. BAs production and consequent accumulation in foods can cause toxicological effects on human health. Plant phenolic compounds are promising alternatives to chemical preservatives and reflect consumers' demand for “green” solutions. In this study, the antimicrobial effect of blackberry (*Rubus fruticosus*) leaves and prickly juniper (*Juniperus oxycedrus*) needles, both as phenolic extracts (PE) and essential oils (EO), were evaluated against *Enterococcus faecium* FC12, a known tyramine-producing strain.

**Methods:**

The growth kinetics in the presence of sub-lethal concentrations of such plant derivatives were modeled (Gompertz equation) and BA production was monitored over time by HPLC. Moreover, flow cytometry (FCM) was used to study the effects of EOs and PEs on cell viability.

**Results:**

The EOs showed a higher antimicrobial effect (especially *R. fruticosus* added at 0.75 mg/ml), determining an initial decrease of culturable cells followed by a recovery, even if with lower growth rates and final cell loads. Different rates of BA formation were observed, with tyramine concentrations ranging from 120 to 160 mg/l after 96 h of incubation, and 2-phenylethylamine was produced in lower amounts, usually after reaching the peak of tyramine. FCM confirmed the higher efficacy of *R. fruticosus* EO that induced cell membrane injury in 93% of the total population. However, complete recovery occurred in the following incubation, demonstrating transient damage.

**Discussion:**

Although further research is required to better investigate this recovery and to assess the suitability of this approach in a real food system, the present study showed the potential antimicrobial activity of plant derivatives, especially *R. fruticosus* EO, against the tyramine-producing *E. faecium* FC12.

## Introduction

Enterococci are lactic acid bacteria (LAB) that can be found in different habitats, and they are highly competitive in harsh conditions due to their relevant salt, pH, and broad temperature range tolerance. For this reason, they are part of the microbiota of several foods of animal origin, such as cheeses and sausages (1–3). However, their presence is controversial (4): on one hand, some enterococci have been described for their probiotic features and capability of producing active bacteriocins against pathogens (3, 5); on the other hand, this genus is known for its antibiotic resistance, which can be transferred to other microorganisms (6, 7), the potential presence of virulence factors (such as cytolysins), aggregation substances, and gelatinase extracellular surface proteins (8, 9).

In addition, enterococci produce biogenic amines (BAs), some basic nitrogenous molecules derived from the decarboxylation of amino acids (10–12). Although these molecules can be formed in controlled amounts by the human body, where they play regulatory roles in different physiological activities, their excessive intake through diet can cause severe symptoms depending on the health status of the consumer (13). Among BAs, histamine and tyramine are those with the most severe acute effects, and they are responsible for the “fish poisoning” symptoms (14) and the “cheese reaction” syndrome (15), respectively. Tyramine is the result of the decarboxylation of tyrosine and may be found in relevant amounts in foods such as cheeses and fermented sausages (16). The tyraminogenic capacity is considered a species characteristic in *Enterococcus faecalis* (8, 13, 17–19), but it is also extremely widespread among *Enterococcus faecium, Enterococcus durans*, and *Enterococcus mundtii* (20, 21). Therefore, strategies that can control the development of these bacteria are crucial points for many fermented products.

In recent years, the antimicrobial activity of plant extracts has been deeply studied, following the consumer demand for “green” preservatives. It is well known that many plant extracts can contain molecules with a relevant antimicrobial activity, which depends, in the first instance, on their composition. Many variables, including seasonal, geographical, and agronomic factors, as well as the mode of extraction and part of the plant, influence the qualitative and quantitative presence of antimicrobial compounds and, in turn, their effectiveness (22, 23).

The Mediterranean maquis is characterized by high plant biodiversity, and many species are known to produce antimicrobial substances. *Rubus fruticosus*, belonging to the family *Rosaceae*, is extremely diffused and popular for blackberry production. Several parts of the plant, such as fruits, leaves, and young shoots, have been widely applied in traditional medicine (24). In addition, their phenolic extract (PE) showed relevant antioxidant activity (25) due to the presence of phenolic acids, flavonoids (anthocyanins, flavonols, and tannins), carotenoid, and organic acids (26). Many of these antioxidant molecules can also exert antimicrobial activity. Aqueous and acetone extracts of *R. discolor* were effective in inhibiting the growth of many bacteria, including *Listeria monocytogenes* (26), while the methanolic extract of stems, leaves, fruits, and roots of *R. fruticosus* was active against *Escherichia coli, Salmonella* Typhi, *Staphylococcus aureus*, with none or scarce effects against yeasts and molds (27). Bioactive phenolics from blackberry pomace were able to reduce *Salmonella* contamination in farm animals (28, 29), and blackberry juice reduced the growth of *L. monocytogenes, E. coli* O157:H7, and *Salmonella* Typhimurium, while it stimulated the growth of LAB such as *Lactiplantibacillus plantarum, Lacticaseibacillus casei*, and *Lacticaseibacillus rhamnosus* (30).

Junipers are another group of plants typical of the Mediterranean maquis. Extracts of *Juniperus communis, Juniperus turbinata, Juniperus deltoides*, and *Juniperus oxycedrus* have been extensively studied for their antioxidant activity (31) and antimicrobial potential (32, 33). Studies have been mainly focused on essential oils (EOs) extracted from leaves and berries, in which α-pinene is usually the major constituent, followed by myrcene, sabinene, limonene, germacrene D, δ-cadinene, and other terpenes and terpenoids (34). Prickly juniper, *J. oxycedrus*, is a typical Mediterranean plant whose EO was effective in inhibiting or reducing the growth of some Gram-positive bacteria. The aqueous extract of *J. oxycedrus* needles did not show an antimicrobial effect, while its methanol extract inhibited, to a different extent, the growth of 57 strains belonging to 24 bacterial species, including *S. aureus, Enterobacter* spp., and *Bacillus* spp (35–37).

This study aimed to evaluate the effects of PEs and EOs obtained from *R. fruticosus* leaves and *J. oxycedrus* needles harvested in the Mediterranean maquis of Croatia (38), on the strain *E. faecium* FC12, a strong tyramine producer (39, 40). The growth dynamics in the presence of a sub-lethal concentration of the plant derivatives were modeled to highlight their antimicrobial effects while the accumulation of tyramine was monitored. Eventually, *E. faecium* cells grown in these conditions were analyzed through flow cytometry to investigate the effect of such plant derivatives on cell viability and culturability.

## Materials and methods

### Enterococcus faecium FC12

The strain *E. faecium* FC12 used in this study and belonging to the Department of Agricultural and Food Sciences (University of Bologna) collection was isolated from a traditional Italian cheese. It was previously described as a high tyramine producer (39, 40). The strain was maintained in BHI medium (Oxoid, Basingstoke, UK) and added with 20% (w/v) glycerol at −80°C until use. Before each experiment, the strain was pre-cultivated two times in BHI medium for 24 h at 30°C.

### Plant derivatives and minimum inhibiting concentration determination

The PEs and EOs used in this experiment, obtained from *R. fruticosus* leaves and *J. oxycedrus* needles, have been previously described and characterized in the study of Barbieri et al. (38).

The *in vitro* antimicrobial activity of these plant derivatives against the target strain was assessed to determine the MIC by broth microdilution method using microtiter plates (Corning Incorporated, NY, USA) according to the method of Arioli et al. (41). In particular, 198 μl of BHI broth inoculated with *E. faecium* FC12 (cell load approximately 6 log CFU/ml) were placed into 200 μl microtiter wells. Then, 2 μl of each plant derivative, previously dissolved in ethanol, was added to each well to obtain final concentrations ranging from 0 to 3 mg/ml. The data were collected after 48 h of incubation at 30°C. The MIC was defined as the lowest concentration of plant derivatives which was able to prevent visible microbial growth in the well.

### Growth kinetics of *E. faecium* FC12 in the presence of plant derivatives

The target microorganism was inoculated in BHI broth with a final concentration (approximately 6 log CFU/ml), and sub-lethal concentrations of plant derivates (50% of MIC) were added to evaluate their effect on the growth kinetics. In particular, 1 mg/ml of extract was used in each condition, except for *R. fruticosus* EO, whose concentration was 0.75 mg/ml. Culturability was assessed by plate counting at a specific time interval during the 96 h incubation at 20°C. Appropriate decimal dilutions were plated into BHI agar medium and incubated at 30°C for 48 h. The analyses were performed in triplicate, and the data obtained from plate counting were modeled with the Gompertz equation as modified by Zwietering et al. (42):


$$\begin{array}{l} y=k+A\cdot e^{-e^{\left[\left(\frac{u_{\max}\cdot e}{A}\right)\cdot \left(\lambda -t\right)+1\;\right]}}, \end{array}$$

where *y* is the cellular load at time t, *k* is a constant representing the initial cellular load (6 log CFU/ml), the parameter *A* represents the difference between the maximum cellular load reached and the initial cell concentration, μ_*max*_ is the maximum log CFU/ml rise rate in exponential phase (h^−1^), and λ is the lag phase duration.

Since, in some samples, an initial decrease in cell counts was observed, a double-peaked Gompertz equation was used to fit also the first part of the curves (43). In this case, the model was as follows:


$$\begin{array}{l} y=k+\left(A_{1}\cdot e^{-e^{\left[\left(\frac{u_{\max1}\cdot e}{A_{1}}\right)\cdot \left(\lambda_{1}-t\right)+1\right]}}\right)+\left(A_{2}\cdot e^{-e^{\left[\left(\frac{u_{\max2}\cdot e}{A_{2}}\right)\cdot \left(\lambda_{2}-t\right)+1\right]}}\;\right), \end{array}$$

where the parameters were defined with the subscript number 1 or 2 to differentiate the first phase of decrease (*A*_1_, μ_*max*1_, and λ_1_) and the second growth phase (*A*_2_, μ_*max*2_, and λ_2_). Data modeling was performed using STATISTICA software (Statsoft Italia, Vigonza, Italy).

### Biogenic amines production

The formation of tyramine and 2-phenylethylamine in the different tested conditions was monitored by collecting samples at a defined time (0, 24, 48, 72, and 96 h). The cell-free supernatants were analyzed using an HPLC Agilent Instrument 1260 Infinity with the automatic injector (G1329B ALS 1260, loop of 20 μl), equipped with a UV detector (G1314F VWD 1260) set at 254 nm. A C18 Waters Spherisorb ODS-2 (150 × 4.6 mm, 3 μm) column was used for the chromatographic separation. Before the injection, the samples were subjected to a dansyl chloride derivatization (Sigma Aldrich, Gallarate, Italy) according to the method of Martuscelli et al. (44). The BAs were expressed as mg/L with reference to the BAs standard calibration curve. All the samples were analyzed in triplicate.

### Flow cytometry (FCM) analysis

To test cell viability, the samples were collected after 24, 48, and 72 h of incubation and analyzed in triplicate with a flow cytometer Accuri C6 (BD Biosciences, Milan, Italy), following the protocol reported by Arioli et al. (41). Before analyses, samples were diluted (if needed) in filtered PBS, and the cells were stained with SYBR-Green I (1X) and propidium iodide (7.5 μM) at 37°C for 15 min. This dual staining allowed to differentiate three sub-populations corresponding to different physiological states: live, injured, and dead cells. The data obtained were analyzed using the BD ACCURITM C6 software version 1.0 (BD Biosciences, Milan, Italy).

## Results and discussion

### Growth kinetics in the presence of plant derivatives

The PEs and the EOs of *R. fruticosus* and *J. oxycedrus*, previously characterized by Barbieri et al. (38), were first tested to define the MIC of the plant derivatives against *E. faecium* FC12. MIC was 1.5 mg/ml for *R. fruticosus* EO and 2 mg/ml for *J. oxycedrus* EO and both the PEs.

**Figure 1:**
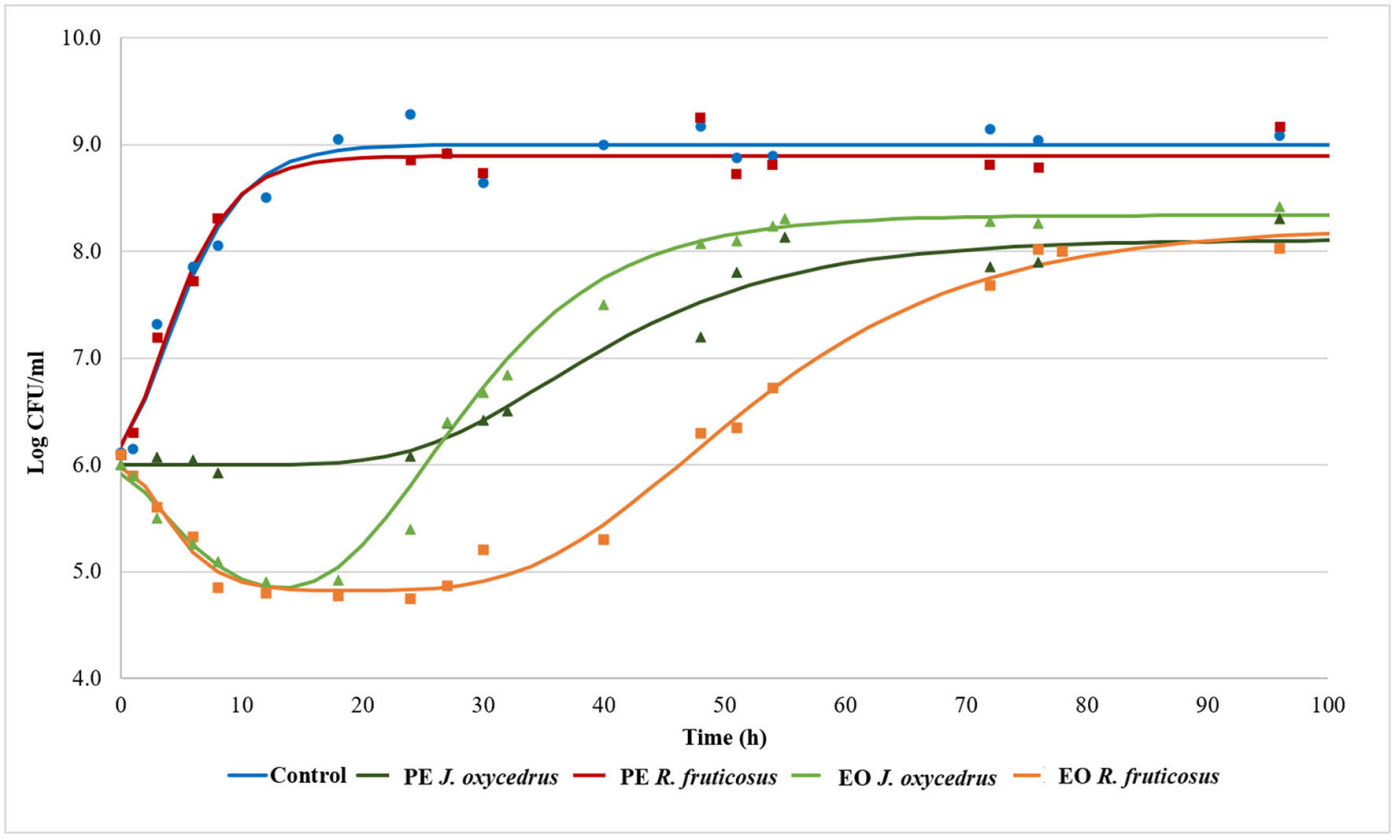
Growth kinetics of *E. faecium* FC12 incubated at 20°C in the presence of different concentrations of plant derivatives: 1 mg/ml of phenolic extracts (PEs) of *J. oxycedrus* needles or *R. fruticosus* leaves, 1 mg/ml of essential oil (EO) of *J. oxycedrus* needles, and 0.75 mg/l of EO of *R. fruticosus* leaves. The experimental data obtained by plate counting are represented with points, while curves are the relative fitted models obtained with the Gompertz equation.

To clearly understand the effects of the EOs and PEs on *E. faecium* FC12, cells were inoculated in BHI and incubated at 20°C. The culture media were added with sub-lethal concentrations of plant derivatives (50% of MIC), aiming to activate a metabolic response due to the stress conditions. The growth dynamics were monitored by plate counting and compared with the control grown in the absence of plant derivatives. Figure 1 reports the experimental points and the corresponding fitted models, which show two different kinetics trends. The control and the samples containing PEs presented a typical growth curve characterized by three distinct steps: lag, exponential, and stationary phases. In contrast, the samples containing EOs showed an initial part in which cells decreased their culturability. After that, cell concentration increased, reaching an exponential phase and a subsequent stationary phase. In the first case, the experimental data were fitted with the classical Gompertz equation (42), while in the second case, a double-peaked Gompertz equation was chosen (38, 43). Table 1 reports the parameters estimated for the models used. In the table, the maximum cell concentration attained according to the models, and some diagnostics of fitting are reported as well.

**Table 1:** *E. faecium* FC12 growth parameters in the presence of different plant derivatives (phenolic extract, PE or essential oil, and EO), estimated by modeling the data from plate counting (log CFU/ml) with the Gompertz equation. The maximum cell concentration attained according to the models, and some diagnostics of fitting are also reported.

**Sample**	**k**	** $A_{1}^{\text{a}}$ **	** $\mu_{max1}^{\text{a}}$ **	** $\lambda_{1}^{\text{a}}$ **	***A_2_***	***μ_*max*2_***	***λ_2_***	**max cell load**	**R**	**loss**
Control	6.03	-	-	-	2.999	0.309	0.11	9.00	0.993	0.157
*J. oxycedrus* PE	5.92	-	-	-	2.108	0.069	24.14	8.11	0.994	0.187
*R. fruticosus* PE	6.08	-	-	-	2.892	0.324	0.08	8.89	0.986	0.377
*J. oxycedrus* EO	5.98	−1.303	−0.128	0.06	3.640	0.160	17.12	8.34	0.995	0.192
*R. fruticosus* EO	6.05	−1.183	−0.174	0.95	3.410	0.095	33.77	8.23	0.994	0.189

^**a**^Parameters estimated only for the double-peaked Gompertz model used when the initial cell concentration decreased before the growth started again.

*E. faecium* FC12 cells in the control rapidly started their exponential phase (the lag phase estimated length was 0.11 h) and reached the maximum cell concentration (approx. 9 log CFU/ml) in < 24 h, with a maximum growth rate (μ_*max*_) of 0.309 h^−1^. The Gompertz parameters estimated for the control and the sample containing *R. fruticosus* PE were similar, and the consequent models were almost completely superimposable, indicating the absence of any antimicrobial effect of this extract. However, the PE of *J. oxycedrus* determined a marked increase of the lag phase (approximately 24 h), a reduced μ_*max*_ value (0.069 h^−1^), and a maximum growth of 8.11 log CFU/ml, resulting in almost 1 log unit lower than the control.

In the previous study, the same PEs were more effective in reducing the growth kinetics of *L. monocytogenes* when used at 50% of the MIC. However, in this case, a relevant inhibition of *E. faecium* was obtained only with *J. oxycedrus* PE, which contains not only high amounts of vanillic acid (10.51 mg/l), apigenin (7.66 mg/l), and rutin (6.95 mg/l) but also gallic acid and *p*-hydroxybenzoic acid (38). The antimicrobial potential of the molecules found in these PEs has been reviewed by Oulahal and Degraeve (45). Rutin, apigenin, and gallic acid showed good antimicrobial activity against *E. faecalis* (46, 47). Taviano et al. (48) observed a remarkable antimicrobial activity against *Enterococcus hirae* of methanolic extracts of *J. oxycedrus* berries, containing rutin, apigenin, cupressoflavone, amentoflavone, and methyl-biflavone, among the others. The *R. fruticosus* PE was rich in rutin, chlorogenic acid, astrignin, and caffeic acid but showed no antimicrobial activity against *E. faecium* under the conditions adopted in this study. Interesting antimicrobial effects on *E. faecalis* were described in an extract from *R. fruticosu*s (rich in ellagic acid), which was absent in the PE used in the present study (49). In addition, Krzepiłko et al. (25) reported the antimicrobial activity of several extracts of *R. fruticosus* buds against the same species. *E. fecalis* showed high susceptibility to quercetin in combination with gallic acid and chlorogenic acid, while insufficient effects were obtained with rutin (50).

The mechanism of action responsible for the antimicrobial activity of these molecules is not completely clear. The literature suggests that some compounds (quercetin, gallic acid, and chlorogenic acid) may induce an imbalance of the redox potential, while others (apigenin and rutin) could interfere with enzymes of DNA replisome or perturbate membrane permeability and functions (50, 51). Recently, Xu et al. (52) observed a depolarization and permeabilization of the membrane and an alteration of the intracellular enzymatic activities in *S*. *aureus* when treated with a phenolic extract from *Taraxacum officinale* that contained rutin, caffeic acid, and chlorogenic acid.

Both EOs determined an initial decrease in *E. faecium* culturability. In the samples containing the *J. oxycedrus* EO, a reduction of more than 1 log unit of the initial population was observed after 10 h, followed by a cell load increase (λ_2_ estimated at 17.12 h). However, in this second phase, the μ_*max*_ (0.160 h^−1^) and the final cell concentration in the stationary phase (8.34 log CFU/ml) were lower when compared to the control.

The main components of the *J. oxycedrus* EO used in this trial were terpenes and terpenoids. Limonene (13.6%), α-pinene (10.8%), and manoyl oxide (8.4%) were the main constituents, followed by 3-carene, α-curcumene, and 4(15),5-muuroladiene (38). Recently, an EO from *Juniperus phoenicea*, whose main constituents were α-pinene, δ-3-carene, and β-caryophyllene, showed good antimicrobial activity against a strain of *E. faecalis*, while Najar et al. (53) reported a variable inhibitory effect of *J. oxycedrus* EOs, depending on the geographical area. Similarly, *E. faecalis* was the most susceptible bacterial species to an EO from *Juniperus horizontalis* leaves, containing *p*-cymene and linalool as the major constituents (54). However, no studies regarding the effects of specific EO constituents on enterococci are available.

*J. oxycedrus* EO and *R. fruticosus* leaves EO reduced the initial culturability of *E. faecium*, although the successive growth started later (λ_2_ estimated at 33.77 h) and slowly (μ_*max*_ 0.095 h^−1^), with a final predicted cell concentration that reached 8.23 log CFU/ml. *R. fruticosus* EO's major constituents were geraniol (13.7%), phytol (4.9%), β-citronellol (4.6%), linalool (4.1%), and β-ionone (3.7%). In addition, the EO contained methyl-salicylate (1.3%) and citral (2.4%) (38). Even if many of these compounds have been studied in relation to their antimicrobial activity (55), no data are reported concerning their effect on enterococci. The antimicrobial effects of terpenes, terpenoids, and phenylpropanoids are strictly dependent on their chemical structure and the presence of specific functional groups. For this reason, the mechanisms of action can be extremely different from one compound to another. However, the first requirement for EO active components relies on the possibility to solubilize in cell membranes which, in many cases, are the major target of their bacteriostatic or bactericidal effects (22, 55–57).

### Biogenic amine production

**Figure 2:**
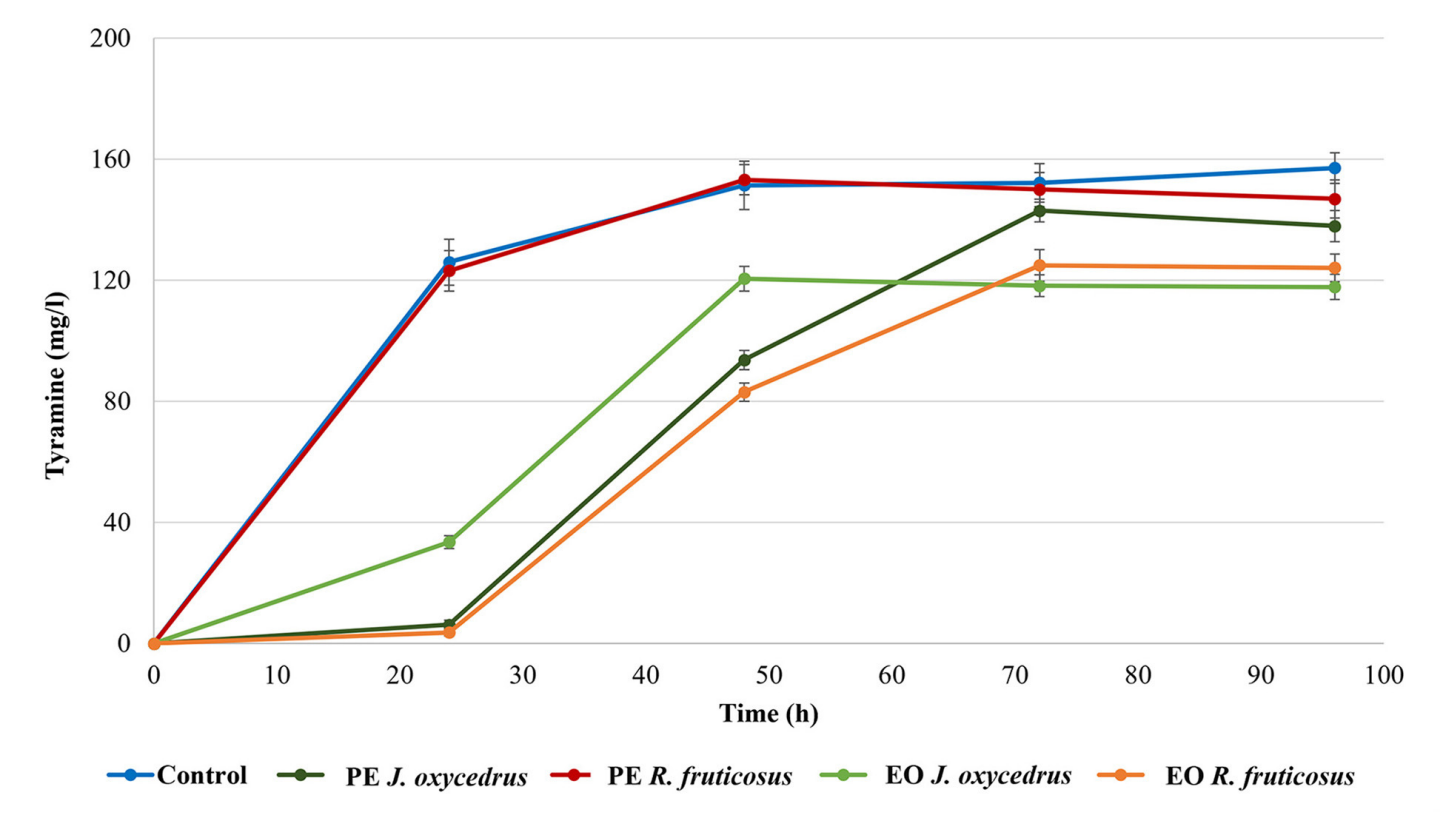
Tyramine production by *E. faecium* FC12 during incubation at 20°C in the presence of different concentrations of plant derivatives. The data are the mean of three independent samples, and standard deviations are reported.

*E. faecium* FC12 has already been studied for its tyrosine decarboxylase activity (39, 40). For this reason, the formation of tyramine was monitored during growth with or without the PEs and EOs (Figure 2). As already observed for the growth curves, the control and sample containing *R. fruticosus* PE showed almost the same behavior. In fact, in both cases, a relevant amount of tyramine (approximately 120 mg/l) was present after 24 h, when the cells had already reached their stationary phase. The concentration slightly increased after 48 h (approximately 150 mg/l) and then remained constant.

The *J. oxycedrus* PE delayed the tyramine formation, with only 6 mg/l detected after 24-h incubation. This concentration increased together with the cell growth, reaching an amount comparable with the control and sample containing *R. fruticosus* PE after 96 h. When *J. oxycedrus* EO was added to the growth medium, tyramine concentration was 33.5 mg/l after 24 h incubation (with an estimated cell concentration of 5.8 log CFU/ml). Then, it increased up to 118.2 mg/l after 48 h (when the maximum cell load of 8.1 log CFU/ml was reached), where it stabilized for the subsequent 48 h. A similar maximum concentration was obtained in the samples containing *R. fruticosus* EO, which, however, showed a slower accumulation kinetic, following the growth curve reported in Figure 1.

**Figure 3:**
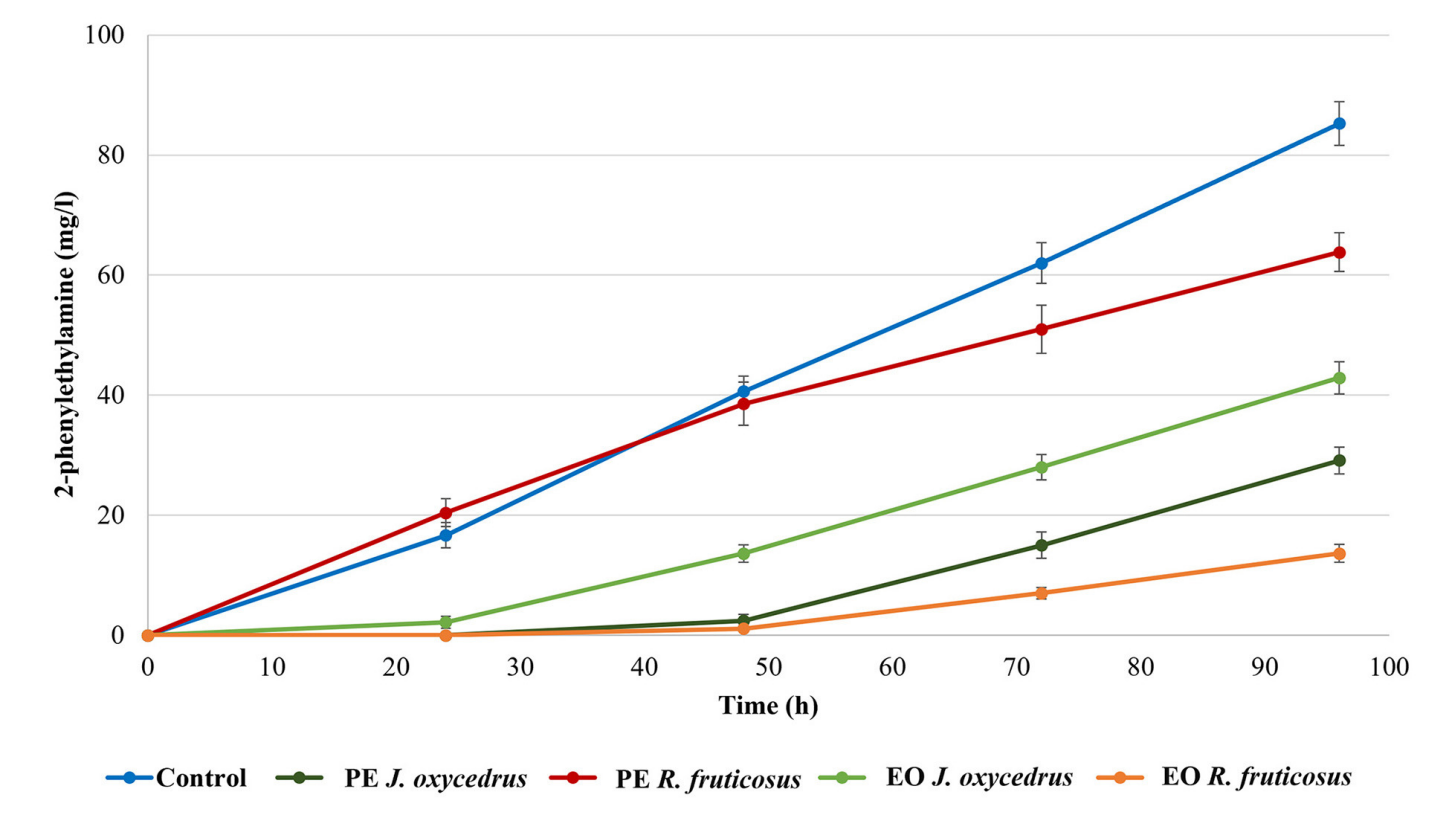
2-phenylethylamine production by *E. faecium* FC12 during incubation at 20°C in the presence of different concentrations of plant derivatives. The data are the mean of three independent samples, and standard deviations are reported.

It is known that, when tyrosine is depleted, the tyrosine decarboxylase of enterococci can use another aromatic amino acid as substrate (phenylalanine), producing 2-phenylethylamine (10, 18, 40). For this reason, this amine was also determined (Figure 3). As expected, it was produced only after tyramine concentration reached its maximum. In the control, 85.3 mg/l of 2-phenylethylamine was found at the end of incubation (96 h). Despite the growth data and tyramine production, in samples containing *R. fruticosus* PE, 2-phenylethylamine accumulation had a trend similar to the control during the first 48 h of incubation, while lower values were measured after 96 h (63.8 mg/l). In contrast, the presence of *J. oxycedrus* EO delayed the production of this amine after 48 h, when tyramine reached its maximum concentration. Even *J. oxycedrus* PE and *R. fruticosus* EO showed the same trend, with the latter EO determining the lowest accumulation of 2-phenylethylamine after 96 h, confirming its antimicrobial effect.

As already observed, detectable tyramine accumulation by *E. faecium* started in the last part of the exponential growth phase, while 2-phenylethylamine was produced by cells in the stationary phase following the depletion of tyrosine (18). The availability of precursors depends on the media composition. Previous experiments showed that *E. faecium* FC12 grown in the same medium (BHI) under optimal conditions (37°C without antimicrobials) determined a final ratio of 3:1 between 2-phenylethylamine and tyramine (40). In this case, after 96 h of incubation, its concentration still had an increasing trend in all the conditions, including the control.

### Effect of plant derivatives on cell viability and culturability

**Figure 4:**
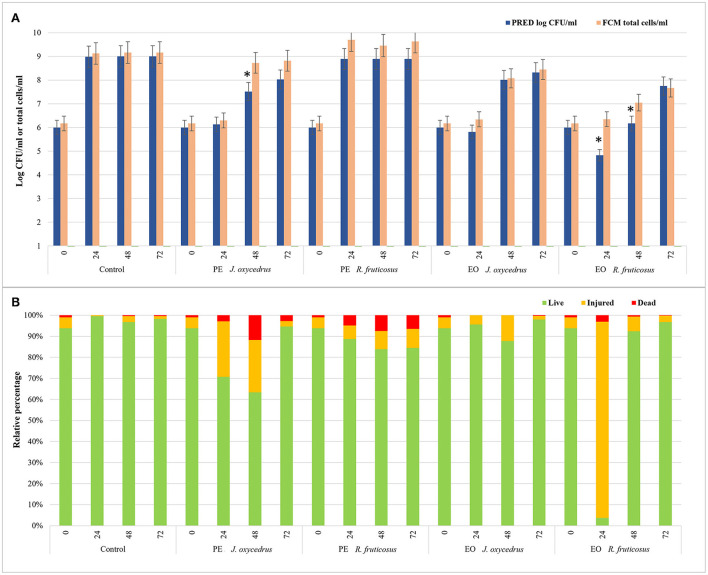
Comparison between the data of cell culturability (expressed as log CFU/ml predicted by the models) and the total cells detected by FCM analysis (log total cells/ml) of *E. faecium* FC12 grown in the presence of different plant derivatives (PE or EO of *J. oxycedrus* and *R. fruticosus*) after 24, 48, and 72 h of incubation at 20°C **(A)**. The presence of an asterisk indicates significant differences between the data of cell culturability and FCM according to Student's *t*-test. For FCM analysis, the relative percentages of live, injured, and dead cells for each condition (as green, yellow, and red bars, respectively) are also reported **(B)**.

To evaluate the impact of EOs and PEs on *E. faecalis* FC12 viability, an FCM protocol (38, 58) was used, and the data obtained after 0, 24, 48, and 72 h of incubation at 20°C were compared with those derived from plate counting. In particular, the total cell number, expressed as a log of cells stained with SYBR-Green I independently of their physiological state, was compared with cell culturability, expressed as log CFU/ml as predicted by the models (Figure 4A). In addition, for each condition and sampling time, the percentages of viable, injured, and dead cells detected after the dual staining with SYBR-Green I and propidium iodide were also reported (Figure 4B).

In the control, culturability (plate counting) and total cells (FCM) did not show significant differences. The viable cells were 94% at time 0 and ranged between 96 and 99% during incubation. Similar values were obtained in the sample containing *R. fruticosus* PE, although a slight decrease in viable cells was observed after 24 and 48 h. With the addition of *J. oxycedrus* PE, the number of culturable and total cells at 24 h did not increase compared to the initial concentration. Even though both values were not significantly different (6.13 log CFU/ml vs. 6.30 total cells/ml), viable cells represented 71% of the total population (corresponding to a concentration of 6.15 log cells/ml), while injured cells were 26%. After 48 h, the culturable cells increased to 7.52 log CFU/ml, whereas the total cell number was 8.73 log total cells/ml. This difference can be related to the presence of viable but non-culturable cells resulting from *J. oxycedrus* PE treatment that induced cell damage. This is supported by the fact that the percentage of cells recognized as live (i.e., with intact cell membrane) still decreased (63%, corresponding to 8.53 log live cells/ml), and dead cells increased (approximately 12% of the total population). At the end of incubation (72 h), total cells and culturable cells were again not significantly different, and a higher number of viable cells (95%) was reached. Therefore, *J. oxycedrus* PE induced temporary cell damage resulting in a slower growth kinetic, namely an increase in lag phase duration and a decrease in growth rate. The same effect with the same PE was already reported in a previous study carried out on *L. monocytogenes* (38).

Data concerning the *J. oxycedrus* EO showed comparable numbers of culturable and total cells during all the incubation. The percentage of live cells ranged from 87.8 to 98.0% log CFU/ml, and the values of viability detected by FCM (log live cells/ml) were substantially the same, indicating an exponential growth between 48 and 72 h.

A different behavior was observed in the presence of *R. fruticosus* EO. The number of total cells detected by FCM did not significantly change between time 0 h and time 24 h (6.17 vs. 6.35 total cells/ml). However, the cells recognized as live corresponded to 3.7% of the total population, while 93.2% resulted as injured. Thus, live cells were 4.92 log cells/ml, a value comparable with the cells detected by plate counting (4.83 log CFU/ml). After 48 h, almost all the cells recovered (92.3%), with 7.02 log live cells/ml compared to the predicted cell concentration that was lower (6.17 log CFU/ml). In fact, at this time, a great part of live cells resulted unculturable with the traditional plate counting. After 72 h, the data from FCM and plate counting were similar, being 7.66 log live cells/ml and 7.75 log CFU/ml, respectively. The effect of *R. fruticosus* EO on *E. faecium* during the first hours of incubation was in line with the results described by Barbieri et al. (38) on *L. monocytogenes*, indicating the ability of this EO to induce relevant cell damage. However, while in their study, damages were lethal up to 48 h (more than 99% of dead cells), those observed in *E. faecium* cells were transient since the bacterium was able to recover within the same time frame. Although the differences observed could depend on the specific constituents of the extracts and their interaction with the bacteria, the exact mechanism of cell recovery activated by *E. faecium* upon exposure to these plant derivatives is still unknown and requires further studies.

## Conclusion

Plant PEs and EOs can have an important role in limiting the growth of spoilage and pathogenic or toxin producer microorganisms in foods. In this study, the response of the tyraminogenic strain *E. faecium* FC12 to sub-lethal concentrations of *J. oxycedrus* needles and *R. fruticosus* leaves plant derivatives was evaluated in terms of cell culturability, cell viability, tyramine, and 2-phenylethylamine production. The PEs were less effective in limiting *E. faecium* kinetics and BA production than the corresponding EOs. In particular, *R. fruticosus* PE did not show any effect, while *J. oxycedrus* PE reduced the growth dynamic of the strain and the resulting tyramine formation. According to FCM analysis, *J. oxycedrus* PE increased the dead or injured cells between 24 and 48 h of incubation. Regarding two EOs, both determined an initial decrease in culturability. In particular, *R. fruticosus* EO was the most effective since many cells were injured already after 24 h. Then they were able to revert to an active metabolic state with lower growth rates and reduced production of tyramine. According to these data, some of the tested plant extracts showed an interesting antimicrobial potential against *E. faecium*. Although the discrepancies between cell culturability and viability must be carefully considered to avoid underestimations of the effective metabolic responses to stress conditions, these “green” solutions can be considered promising candidates for the microbial stabilization and shelf-life extension of foods. Indeed, further studies are in progress, and they want to assess the suitability of these approaches to controlling enterococci, as well as other spoilage or pathogen microorganisms, in animal-based foods (e.g., meat products).

## Data availability statement

The raw data supporting the conclusions of this article will be made available by the authors, without undue reservation.

## Author contributions

GT, CM, and FG: conceptualization and writing–original draft preparation. FB and DG: literature data collection. SL, DG, and FB: microbiological and chemical analyses. CM: FCM analyses. DG, FÖ, and VŠ: writing–review and editing. All authors have read and approved the submitted version of the manuscript.
